# The complete mitochondrial genome of *Lixus subtilis* Boheman, 1835 (Coleoptera, Curculionidae) and its phylogenetic implications

**DOI:** 10.1080/23802359.2021.2008278

**Published:** 2021-12-10

**Authors:** Kun Xing, Kang Chen, Xiao-jun Zhao, Fei Zhao

**Affiliations:** Shanxi Key Laboratory of Integrated Pest Management in Agriculture, College of Plant Protection, Shanxi Agricultural University, Taiyuan, China

**Keywords:** *Lixus subtilis*, mitochondrial genome, phylogeny

## Abstract

The first complete mitochondrial genome of *Lixus subtilis* Boheman is reported in this study. The circular genome is 15,223 bp long, including a standard set of 21 transfer RNAs (tRNAs), 2 ribosomal RNAs (rRNAs), 13 protein-coding genes, and a non-coding control region. The *trnI* gene was not found in the *L. subtilis* mitogenome. All tRNAs had the typical cloverleaf structure, except for *trnS1*, which lacked the dihydrouridine arm. The phylogenetic tree of 13 Curculionidae species based on the concatenated nucleotide sequences of complete mitochondrial genomes strongly supported that *L. subtilis* is closely related to Curculioninae and Molytinae.

*Lixus subtilis* Boheman, 1835 (Coleoptera: Curculionidae) is widely distributed (Volovniket al. 2015; Davidian et al. 2017) and a primary pest of beets, amaranth, and gray vegetables (Davidian et al. 2017). In recent years, *L. subtilis* has had outbreaks in the quinoa growing areas of Shanxi and Beijing, China, causing large crop losses (Zhang et al. 2017). 30 adult specimens of *L. subtilis* were collected from the quinoa field in Supo Village, Supo Township, Jingle County, Shanxi Province, China (112.2°E, 38.4°N) on 10 May 2018. One specimen was deposited in the College of Plant Protection, Shanxi Agricultural University, Taiyuan, China (Kun Xing, xingkun1215@126.com) under the accession no. YFMgnjP2018393.

Thirty specimens were used in the mitogenomic studies. The complete mitogenome of *L. subtilis* is a representative circular DNA molecule with a length of 15,223 bp (GenBank accession no. MW413392). Thirteen protein-coding genes (PCGs), 21 transfer RNA genes (tRNAs), the large and small ribosomal RNA unit genes (*rrnL* and *rrnS*), and a large non-coding region (putative control region) were contained by this mitogenome. In Coleoptera, the order and orientation of the mitochondrial genomes have been retained from the ancestral gene order, apart for the tRNA gene, which may be deleted or rearranged in some species (Timmermans and Vogler 2012). The *trnI* was not found in the *L. subtilis* mitogenome, as observed in *Eucryptorrhynchus chinensis* (Oliver, 1790) and *Naupactus xanthographus* (Germar, 1824) (Tang et al. 2017; Yang et al. 2018). The nucleotide composition of *L. subtilis* was significantly biased: A, G, C, and T accounted for 40.2%, 9.6%, 14.7%, and 35.5%, respectively; A + T contents totaled 75.7%. In this genome, the GC-skew and AT-skew were −0.209 and 0.063, respectively. Gene overlaps had a total of 45 bp and were present in ten gene junctions. The largest gene overlap (−17 bp) was present between *trnF* and *nad5,* in which intergenic spacers totaling 53 bp appeared in 11 positions and ranged in size from 1 to 18 bp. The control region had A + T content of 72.0% with 571 bp in length and was present between the *rrnS* and *trnQ* genes.

It was predicted that all 21 tRNAs had typical cloverleaf secondary structures, but the gene *trnS1* lacked a stable DHU arm. This result was as the same as those reported in other insect mitogenomes (Yuan et al. 2016). The *rrnL* gene was located between the *trnL1* and *trnV* genes, and the *trnV* and *rrnS* genes were located between the *trnV* gene and the control region. The *rrnL* gene had an A + T content of 81.3% and a length of 1291 bp. The *rrnS* gene was 813 bp in length and had A + T content of 76.3%. Eleven PCGs had a typical ATN codon. PCGs *nad2*, *nad4*, *nad4l*, and *cob* started with ATG; *cox1*, *cox2*, *atp8*, *cox3*, and *nad6* started with ATT; and *atp6* and *nad3* started with ATA. However, *nad5* and *nad1* started with GTG and TTG, respectively. Ten PCGs terminated with TAA and two terminated with TAG (*atp8* and *nad1*), whereas one terminated with an incomplete stop codon TA (*nad4*).

For the phylogenetic analysis, the nucleotide sequences of complete mitochondrial genomes from 13 species (Curculionidae) and outgroups from *Acyrthosiphon pisum* (Harris, 1776) (Hemiptera, Aphididae) were used. This phylogenetic analysis was performed using the maximum likelihood (ML) method with 1,000 bootstrap replicates using MEGA-X and via Bayesian inference (BI) using MrBayes (Ronquist and Huelsenbeck 2003; Kumar et al. 2018). There was strong support for clustering of *L. subtilis* with Curculioninae and Molytinae (Figure 1), indicating that *L. subtilis* is more closely related to Curculioninae and Molytinae than other subfamilies.

**Figure 1.**
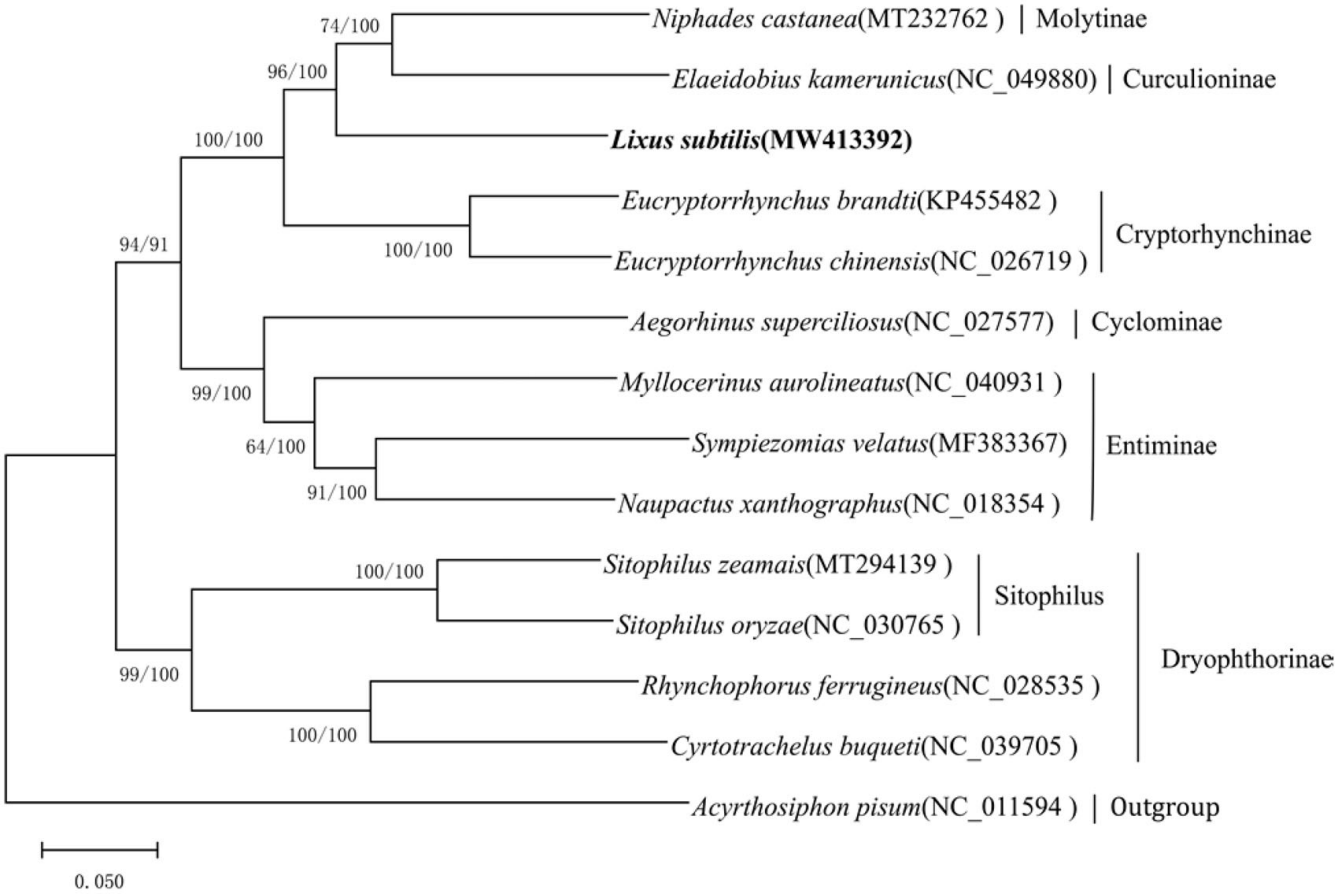
Phylogenetic relationships of 13 Curculionidae, including *Lixus subtilis*, based on mitochondrial genome sequences using ML and BI methods. The numbers beside the nodes are bootstrap values (ML) and posterior probabilities (BI), respectively.

## Data Availability

The genome sequence data that support the findings of this study are openly available in GenBank of NCBI at https://www.ncbi.nlm.nih.gov/genbank under the accession no. MW413392. The associated BioProject, SRA, and Bio-Sample numbers are PRJNA757215, SRR15603403, and SAMN20955048, respectively.
